# Sakuranetin Inhibits Inflammatory Enzyme, Cytokine, and Costimulatory Molecule Expression in Macrophages through Modulation of JNK, p38, and STAT1

**DOI:** 10.1155/2016/9824203

**Published:** 2016-09-07

**Authors:** Ki-Young Kim, Hee Kang

**Affiliations:** ^1^Department of Genetic Engineering, College of Life Science and Graduate School of Biotechnology, Kyung Hee University, Yongin, Republic of Korea; ^2^Graduate School of East-West Medical Science, Kyung Hee University, Yongin, Republic of Korea

## Abstract

Sakuranetin is flavonoid phytoalexin that serves as a plant antibiotic and exists in* Prunus* and several other plant species. Recently, we identified the anti-inflammatory effect of* Prunus yedoensis* and found that there were few studies on the potential anti-inflammatory activity of sakuranetin, one of the main constituents of* Prunus yedoensis*. Here, we isolated peritoneal macrophages from thioglycollate-injected mice and examined whether sakuranetin affected the response of the macrophages in response to lipopolysaccharide (LPS) plus interferon- (IFN-) *γ* or LPS only. Sakuranetin suppressed the synthesis of iNOS and COX2 in LPS/IFN-*γ* stimulated cells and the secretion of TNF-*α*, IL-6, and IL-12 in LPS stimulated cells. The surface expression of the costimulatory molecules, CD86 and CD40, was also decreased. Among the LPS-induced signaling molecules, STAT1, JNK, and p38 phosphorylation was attenuated. These findings are evidence that sakuranetin acts as anti-inflammatory flavonoid and further study is required to evaluate its* in vivo* efficacy.

## 1. Introduction

Inflammatory responses are protective against further tissue damage and help to repair wounds. Inflammatory stimuli not only are confined to microbes, but also include endogenously generated substances, as seen with gout and atherosclerosis [1]. Inflammation should be self-limiting, but when this capacity is impaired, the response will result in continued tissue destruction. The reasons for the chronicity of inflammation include microbes that evade the immune system, accumulating metabolic or cellular byproducts, and autoimmune diseases generated by unknown causes.

Depending on the time required to initially respond, the site of first contact with the antigen, and the ability to acquire memory, the immune system is divided into innate and adaptive systems. Cells that belong to the innate immune system confront the antigens and respond to them immediately but do not acquire memory. On the other hand, adaptive immune cells make first contact with antigens in secondary lymphoid tissue such as lymph nodes, which explains why they take time to respond, and acquire memory, letting the cells mount a faster response to the next exposure of the antigen. Macrophages belong to the innate immune system but present antigens to T cells, acting as a bridge between the innate and adaptive immune systems. Generally, macrophages are the first sensor to detect and react to foreign microbes and, when necessary, recruit other circulating white blood cells to the site [2]. During inflammatory responses, macrophages recognize the presence of the causative agent through pattern recognition receptors such as toll-like receptor (TLR) and activate the NF-*κ*B pathway and mitogen-activated protein kinases (MAPK) pathway, terminating in the expression of inflammatory enzymes such as inducible nitric oxide synthases (iNOS) and cyclooxygenase- (COX-) 2 and inflammatory cytokines such as tumor necrosis factor- (TNF-) *α*, interleukin- (IL-) 6, and IL-12 [3]. In addition, macrophages upregulate the surface expression of costimulatory molecules such as CD80/CD86 and CD40 in order to form stable contacts with T cells. Thus, the above molecules are targets of anti-inflammatory agents for the control of chronic inflammation.

Sakuranetin is flavonoid phytoalexin that serves as a plant antibiotic [4] and exists in the* Prunus* species,* Baccharis* species,* Betula* species, and rice [5]. Recently, we identified the* in vitro* and* in vivo* anti-inflammatory effects of* Prunus yedoensis* bark [6, 7] and found that reports on the anti-inflammatory mechanism of sakuranetin, one of the main constituents of* Prunus yedoensis* bark, were scarce. A literature search on sakuranetin showed that it inhibits chemically induced edema in mice [8] and alleviates the allergen-induced lung injury model through control of NF-*κ*B [9]. Here, we sought to investigate the anti-inflammatory activity of sakuranetin and its mechanism using lipopolysaccharide (LPS) plus interferon- (IFN-) *γ* or LPS stimulated macrophage model.

## 2. Materials and Methods

### 2.1. Animals

Seven-week-old male BALB/c mice (Samtaco, Osan, Korea) were purchased and kept in a temperature- and humidity-controlled, pathogen-free animal facility at Kyung Hee University. The mice were provided with standard mouse chow and water* ad libitum* in accordance with the Guide for the Care and Use of Laboratory Animals issued by the United States National Research Council (1996), and the protocol (KHUSASP(GC)-10-001) was approved by the Kyung Hee University Institutional Animal Care and Use Committee.

### 2.2. Cell Culture

Mice were injected intraperitoneally with 2 mL of 3.5% sterile thioglycollate solution (BD, Sparks, MD, USA). Three days later, mice were sacrificed by cervical dislocation and macrophages were isolated by peritoneal lavage with cold DMEM. After centrifugation, cells were resuspended in DMEM with 10% fetal bovine serum (FBS; Hyclone, Utah, USA) and 1% penicillin-streptomycin and incubated overnight in a humidified atmosphere of 5% CO_2_ at 37°C. After nonadherent cells were removed, cells were seeded for subsequent assays.

### 2.3. Viability Assay

Cells were seeded in quadruplicate in 96-well plates and stimulated for 24 h at increasing concentrations of sakuranetin (Sigma, St. Louis, MO, USA). Cell viability was determined using the MTS (3-(4,5-dimethylthiazol-2-yl)-5-3(carboxymethoxyphenyl)-2-(4-sulfophenyl)-2H-tetrazolium) reduction method (CellTiter 96 One Solution Cell Proliferation Assay Kit, Promega, Madison, WI, USA), based on the measurement of mitochondrial respiration in living cells. Optical density was measured at 490 nm with a microplate reader (Molecular Devices, Sunnyvale, CA, USA).

### 2.4. Measurement of Nitrites

Cells were stimulated with 1 ng/mL of recombinant IFN-*γ* (BD Pharmingen, San Diego, CA, USA) and 100 ng/mL LPS (Sigma) in the presence of sakuranetin or 1 *μ*M dexamethasone (Sigma) for 16 h. Supernatant was obtained for the evaluation of nitrite levels using the Griess Reagent System (Promega). The absorbance at 550 nm was measured with the microplate reader.

### 2.5. Cytokine Measurement

Cells were cultured with 100 ng/mL LPS and sakuranetin for 6 h or 24 h. The cytokine levels from appropriately diluted supernatants were measured by ELISA according to the manufacturer's protocol (BD Pharmingen).

### 2.6. Western Blotting

To detect iNOS and COX-2, cells were stimulated with LPS/IFN-*γ* in the presence of sakuranetin for 16 h. To detect phospho-STAT1, cells were pretreated with sakuranetin for 1 h and then stimulated with LPS for 3 h. To detect I*κ*B*α* and phospho-MAPK, cells were pretreated with sakuranetin for 1 h and then LPS was added for 15 min. Total cell extracts were prepared by resuspending the cells in lysis buffer (50 mM Tris-HCl, pH 7.5; 150 mM NaCl; 1 mM EDTA; 20 mM NaF; 0.5% NP-40; and 1% Triton X-100) containing a phosphatase inhibitor cocktail (Sigma) and an Xpert protease inhibitor cocktail (GenDEPOT, TX, USA). Protein concentration was determined using the Bradford assay. Cell extracts were separated on an 8% or 10% sodium dodecyl sulfate-polyacrylamide gel and were transferred to polyvinylidene fluoride membrane. The membranes were blocked with 5% skim milk in Tris-buffered saline with 0.1% Tween 20 (TBST) for 1 h and then incubated overnight at 4°C with iNOS, I*κ*B*α*, tubulin, or GAPDH (Santa Cruz Biotechnology, Santa Cruz, CA, USA), phospho-STAT1, STAT1, phospho-JNK, JNK, phospho-p38, p38, phospho-ERK, or ERK (Cell Signaling Technology, CA, USA) diluted at 1/1000 in 5% skim milk in TBST. The blots were washed with TBST and incubated for 1 h with anti-rabbit horseradish peroxidase-conjugated antibody (diluted at 1 : 5000 in 5% skim milk in TBST). Protein bands were detected with EzWestLumi plus (ATTO, Japan) and analyzed using an EZ-Capture MG (ATTO). The band density of each protein was quantified using ImageJ software and normalized with internal control.

### 2.7. Flow Cytometry

Cells were washed twice in cold phosphate buffered saline (PBS) and resuspended at 1 × 10^6^ cells/mL in FACS buffer (PBS/0.1% NaN_3_/1% FBS). Cells were blocked with rat anti-mouse CD16/CD32 (BD Pharmingen) at 4°C for 5 min and then stained for 30 min with FITC-conjugated anti-mouse-CD40 and PE-conjugated anti-mouse CD86 (BD Pharmingen) on ice in the dark. For isotype controls, FITC-conjugated rat IgG2a *κ* or PE-conjugated rat IgG2a *κ* (BD Pharmingen) was used. The cells were washed twice and resuspended in FACS buffer. Ten thousand cells were collected for each sample and analyzed on a Navios Flow Cytometer (Beckman Coulter, Brea, CA, USA). The data were analyzed with Kaluza software.

### 2.8. Statistical Analysis

Statistical analysis was performed using Student's *t*-test or ANOVA followed by the SNK test using IBM Statistics SPSS version 22. *P* values less than 0.05 were considered significant.

## 3. Results

### 3.1. Effect of Sakuranetin on Cytotoxicity

**Figure 1:**
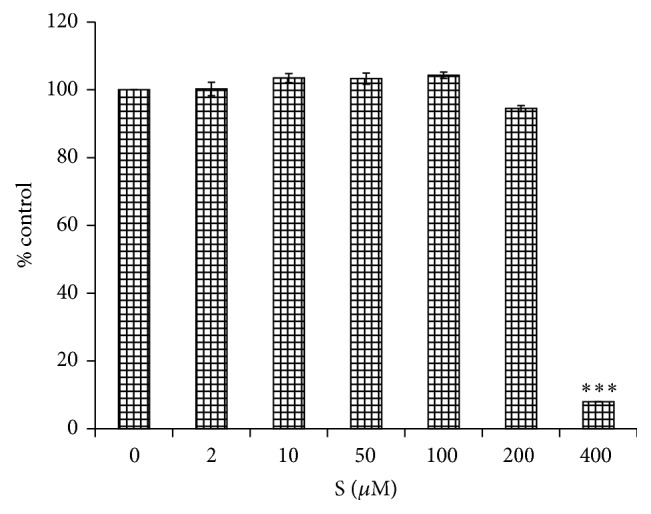
Effects of sakuranetin on cell viability. Mouse peritoneal macrophages were cultured with sakuranetin for 24 h and cell viability was determined using the MTS assay. Data are represented as a percentage of control cells (0 *μ*g/mL) (*n* = 4). ^*∗∗∗*^ *P* < 0.005 versus control.

First, we sought to determine the noncytotoxic range of sakuranetin using the MTS assay. A culture of peritoneal macrophages incubated with 200 *μ*M for 24 h resulted in no effect on cell viability, but cells incubated with 400 *μ*M sakuranetin showed a rapid decrease in number (Figure 1). Based on these results, subsequent assays were performed at no higher than 100 *μ*M.

### 3.2. Effect of Sakuranetin on the NO Production and the Expression of iNOS and COX-2 in LPS/IFN-*γ* Stimulated Cells

**Figure 2:**
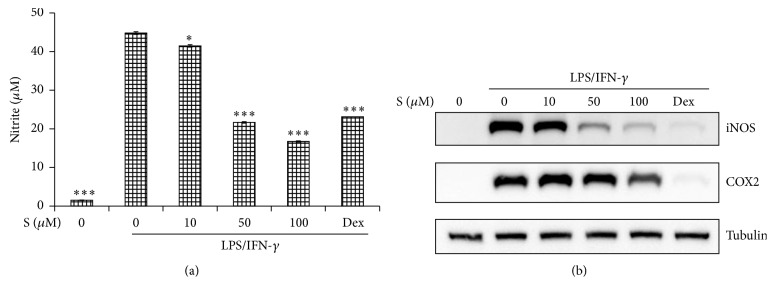
Effects of sakuranetin on the release of nitric oxide and the synthesis of inducible NO synthase (iNOS) and COX2 in LPS/interferon- (IFN-) *γ* stimulated cells. Mouse peritoneal macrophages were stimulated with IFN-*γ* and LPS in the presence of sakuranetin for 24 h. (a) NO in the supernatant was detected by the Griess reaction. Dexamethasone (1 *μ*M) was treated as a reference drug. Data are expressed as mean ± SD (*n* = 3), ^*∗*^ *P* < 0.05 and ^*∗∗∗*^ *P* < 0.005 versus controls (LPS/IFN-*γ* treated cells). (b) The expression of iNOS and COX2 protein was analyzed by Western blotting using tubulin as an internal control. One of the three independent experiments is shown.

When LPS and IFN-*γ* are coadministered to macrophages, full production of NO occurs [10]. In an effort to explore the anti-inflammatory potential of sakuranetin, we first measured the level of NO in the supernatant from activated macrophages. Sakuranetin was added to the cells simultaneously with those inflammatory stimuli. Dexamethasone (1 *μ*M) was used as a reference chemical. Since NO has a short half-life [11], the level of nitrite, another product obtained during NO synthesis, was measured using the colorimetric method. A reduction in NO release by sakuranetin occurred in a dose-dependent manner (Figure 2(a)). Then we examined whether this reduction was due to iNOS protein inhibition. The suppressive effect of sakuranetin on iNOS protein was dose-dependent, as measured by Western blotting (Figure 2(b)). We also measured the level of COX-2 protein from the same cells. A higher concentration (100 *μ*M) of sakuranetin was required to inhibit COX-2 protein than that used in iNOS protein synthesis.

### 3.3. Effect of Sakuranetin on Soluble Inflammatory Cytokine Expression

**Figure 3:**
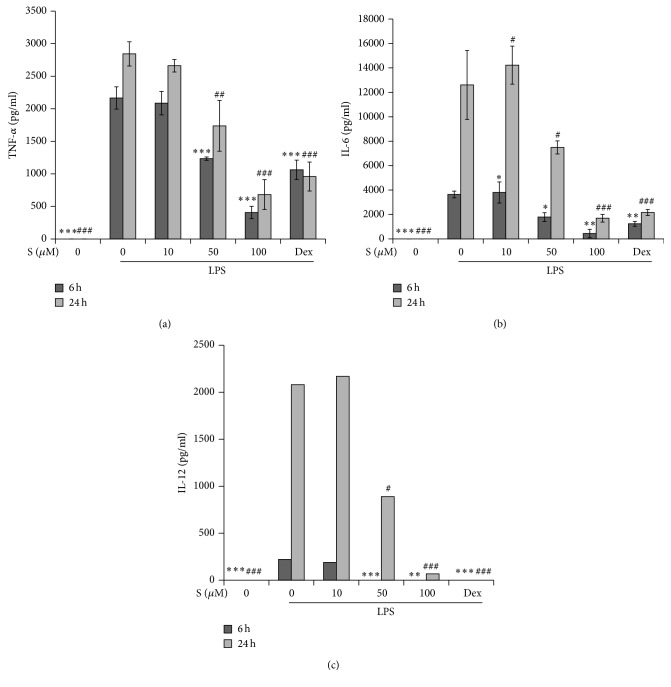
Sakuranetin decreases the secretion of tumor necrosis factor- (TNF-) *α*, interleukin- (IL-) 6, and IL-12. Mouse peritoneal macrophages were stimulated with LPS in the presence of sakuranetin or dexamethasone (1 *μ*M) for 6 h or 24 h, and the levels of TNF-*α* (a), IL-6 (b), and IL-12 (c) in the supernatant were analyzed by ELISA. Data are expressed as mean ± SD (*n* = 3). ^*∗*^ *P* < 0.05, ^*∗∗*^ *P* < 0.01, and ^*∗∗∗*^ *P* < 0.005 versus controls (cells treated with LPS for 6 h); ^  #^ *P* < 0.05, ^##^ *P* < 0.01, and ^###^ *P* < 0.005 versus controls (cells treated with LPS for 24 h).

We stimulated macrophages with LPS in the presence of sakuranetin for 6 h or 24 h, and the levels of TNF-*α*, IL-6, and IL-12 were measured by ELISA. From a gap in cytokine level at 6 h and 24 h, it was clear that the peak of TNF-*α* secretion was earlier than those of IL-6 and IL-12 (Figure 3). Sakuranetin at 50 and 100 *μ*M and dexamethasone decreased the levels of all the cytokines tested at each time point.

### 3.4. Effect of Sakuranetin on Surface Costimulatory Molecules

**Figure 4:**
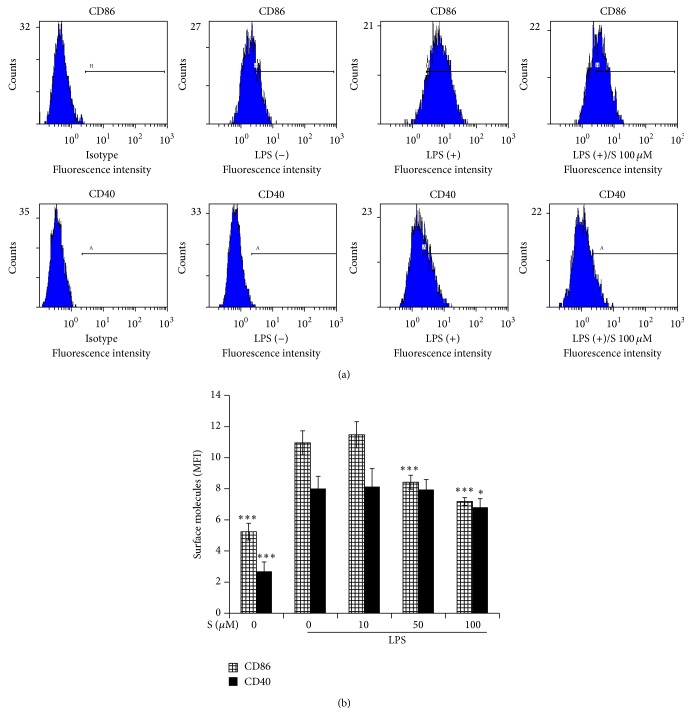
Sakuranetin decreases the expression of costimulatory molecules. Mouse peritoneal macrophages were stimulated with LPS in the presence of sakuranetin for 24 h. The cells were stained for FITC-conjugated CD40 antibody or PE-conjugated CD86 antibody and analyzed using flow cytometry. (a) Representative histograms are shown. (b) The value of mean fluorescence intensity (MFI) was analyzed and data are expressed as mean ± SD (*n* = 4). ^*∗*^ *P* < 0.05 and ^*∗∗∗*^ *P* < 0.005 versus controls (cells treated with LPS only).

We analyzed the influence of sakuranetin on the surface expression of costimulatory molecules CD86 and CD40 using flow cytometry. Treatment of macrophages with LPS increased the mean fluorescence intensity (MFI) of CD86 from 5.24 to 10.95 and that of CD40 from 2.69 to 8.01 (Figure 4). The MFI value of CD86 was decreased in a dose-dependent manner with 75% and 65% of control cells at 50 and 100 *μ*M, respectively. CD40 expression was decreased by 15% at 100 *μ*M compared with controls.

### 3.5. Effects of Sakuranetin on I*κ*B*α* Degradation and MAPK Activation

**Figure 5:**
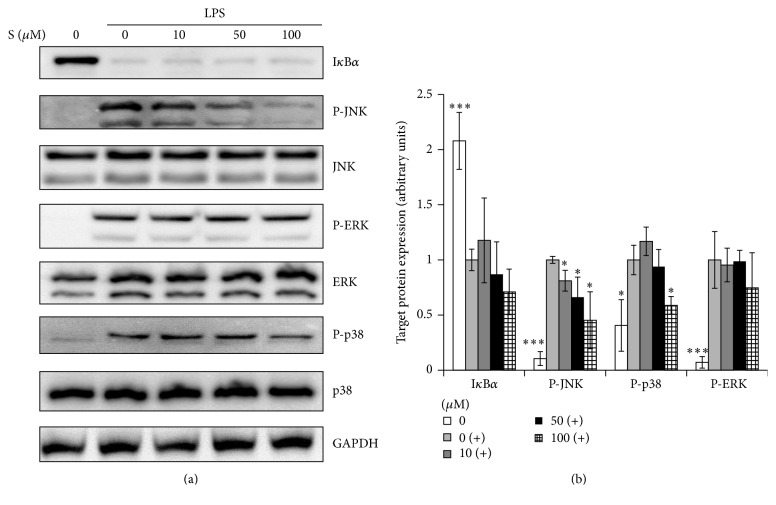
Effects of sakuranetin on LPS-induced I*κ*B*α* degradation and activation of JNK, ERK1/2, and p38. Cells were pretreated with sakuranetin for 1 h and then stimulated with LPS for 15 min. Total protein was extracted and assayed for signaling molecules by Western blotting. GAPDH was used as an internal control. (a) One of the three experiments is shown. (b) The band density of each phosphorylated protein was normalized with GAPDH. ^*∗*^ *P* < 0.05 and ^*∗∗∗*^ *P* < 0.005 versus controls (cells treated with LPS only).

The inflammatory gene expression initiated by LPS/TLR4 signaling depends on the NF-*κ*B and MAPK signaling pathways. I*κ*B*α* plays a critical role in the control of NF-*κ*B signaling by preventing it from migrating to the nucleus [12]. Sakuranetin had no effect on I*κ*B*α* degradation at 15 min (Figure 5). We examined the influence of sakuranetin on the activation of MAPK (p38, JNK, and ERK). The expression of phosphorylated JNK was attenuated in a dose-dependent manner while that of phosphorylated p38 was suppressed at 100 *μ*M (Figure 5).

### 3.6. Effect of Sakuranetin on STAT1 Activation

**Figure 6:**
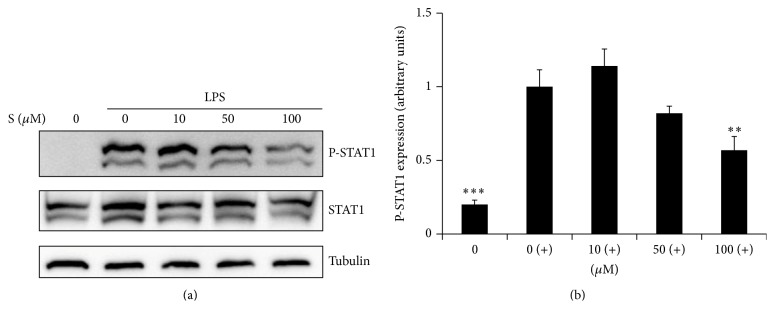
Sakuranetin attenuates LPS-induced STAT1 phosphorylation. Cells were pretreated with sakuranetin for 1 h and then stimulated with LPS for 3 h. Total protein was extracted and assayed for phosphorylated STAT1 by Western blotting. Tubulin was used as an internal control. (a) One of the three experiments is shown. (b) The band density of phosphorylated STAT1 was normalized with tubulin. ^*∗∗*^ *P* < 0.01 and ^*∗∗∗*^ *P* < 0.005 versus controls (cells treated with LPS only).

STAT1 is a critical signaling molecule for the expression of IFN-mediated genes such as iNOS [13]. STAT1 activation elicited by LPS is weak and delayed relative to addition of IFN-*γ* [14]. We found that LPS alone induced STAT1 activation (Figure 6). The expression of phospho-STAT1 was suppressed at 100 *μ*M of sakuranetin (Figure 6).

## 4. Discussion

Flavonoids are the most abundant polyphenols with a C6-C3-C6 backbone structure. Flavonoids exist in vegetables and fruits and are suggested to account for some of the known biological functions of herbal plants. In particular, anti-inflammatory actions of flavonoids are summarized into antioxidant activity and modulation of arachidonic acid metabolizing enzymes and inflammatory molecules [15]. Flavonoids can inhibit enzymes that produce oxygen-derived free radicals and directly reduce those oxidants, thus protecting cells from oxidative damage [16]. Also, flavonoids are reported to interfere with generation of inflammatory eicosanoids and cytokines at multiple levels.

In the case of eicosanoids, arachidonic acids are released from membrane phospholipids by phospholipase A2 and then further converted into prostaglandins by cyclooxygenase or leukotrienes by lipoxygenase. Sakuranetin was proven to be a potent inhibitor of leukotriene B_4_ production in rat neutrophils through modulation of 5-lipoxygenase activity, with IC_50_ of 9 *μ*M, but it failed to inhibit prostaglandin E_2_ production at 25 *μ*M in macrophages [8]. Corroborating the prior study, a higher concentration (100 *μ*M) of sakuranetin was required to inhibit COX2 synthesis.

Although sakuranetin was not very effective in the modulation of the COX2 pathway, this flavonoid was very potent in the suppression of NO and iNOS. NO is produced from arginine and oxygen by NO synthase. While NO is constitutively produced in neurons and endothelial cells by neuronal NO synthase and endothelial synthase, respectively, the expression of iNOS in macrophages is inducible in the presence of inflammatory stimuli such as LPS and cytokines [17]. The role of NO depends on the cell type. For example, neuronal NO is a neurotransmitter, endothelial NO functions as a vasodilator and antiplatelet agent, and NO in macrophages is microbicidal. However, when NO is excessively produced by iNOS, it is harmful to cells because of its toxic effects produced by reacting to the thiol group of proteins, nucleic acids, unsaturated lipids, divalent cations, and other reactive oxygen species. STAT1 is necessary for NO production in macrophages in response to LPS plus type I IFN (IFN-*α*,*β*) or type II IFN (IFN-*γ*) [13]. STAT1 activation occurs when type I IFN or type II IFN binds to its receptor. Since the promoters of the iNOS gene have binding sites of NF-*κ*B and STAT1, maximal expression of iNOS in mouse macrophages can be achieved with stimulation of LPS and IFN-*γ* [17]. Since sakuranetin had no effect on I*κ*B*α* degradation, its effect on iNOS protein is unlikely to involve NF-*κ*B signaling. Rather, the reduction of iNOS by sakuranetin partly appears to depend on the inhibition of STAT1 activity.

TNF-*α*, IL-6, and IL-12 are the major cytokines released by activated macrophages during an early inflammatory response. One of their roles is to induce differentiation and migration of other immune cells, bridging innate and adaptive immunity. Importantly, IL-12 is a cytokine that determines the differentiation of CD4 T cells into IFN-*γ*-producing T helper cells, which further activate macrophages [18]. Sakuranetin decreased the levels of these cytokines in a dose-dependent manner. MAPK and their downstream effector proteins are involved in the modulation of transcription factor required for inflammatory genes or the stability of the mRNAs [3]. Several studies have demonstrated that pharmacological inhibitors of JNK, ERK1/2, or p38 suppress LPS-induced iNOS, TNF-*α*, IL-6, and IL-12 gene expression [19–21]. It is possible that sakuranetin interferes with downregulation of the cytokines and partly of iNOS via its inhibitory effect on JNK and p38 activation.

CD86 and CD40 are often used as activation markers of macrophages [22]. The mode of these costimulatory molecules is contact-dependent. Contact between CD86 on macrophages and CD28 on T cells or between CD40 on macrophages and CD40 ligand on activated T cells enhances each cell's own activity. For example, CD40 itself enhances macrophages' function by increasing the expression of NO, TNF-*α*, IL-6, and CD86 [23]. Inhibition of these costimulatory molecules is expected to attenuate interactions between macrophages and T cells observed in chronic inflammatory responses. The upregulation of CD86 and CD40 depends on NF-*κ*B and STAT1 [13, 24]. The inhibitory effect of sakuranetin on these costimulatory molecules seems to be attributed to its attenuation of STAT1. Interestingly, the decrease of CD40 and COX2 requires a higher concentration than was necessary to induce the decrease of CD86, cytokines, and iNOS. LPS and its sensor TLR4 use two separate adaptor molecules, Myd88 and TRIF, respectively. Myd88 is responsible for early NF-*κ*B and MAPK activation or inflammatory genes, while TRIF is associated with late activation of NF-*κ*B and MAPK and adaptive immune responses such as IFN-inducible genes [25]. Therefore, certain inflammatory proteins are Myd88- and TRIF-codependent while others are TRIF-dependent only [26]. It is possible that sakuranetin differentially acts in these signaling pathways.

## 5. Conclusions

Taken together, these findings are evidence that sakuranetin acts as an anti-inflammatory flavonoid by inhibiting the expression of iNOS, TNF-*α*, IL-6, and IL-12 and by downregulating the surface expression of costimulatory molecules. Some of the cellular signaling mechanisms regulated by sakuranetin are based on its modulation of JNK, p38, and STAT1 phosphorylation.

## References

[1] Zhang X., Mosser D. M. (2008). Macrophage activation by endogenous danger signals. *The Journal of Pathology*.

[2] Mosser D. M., Edwards J. P. (2008). Exploring the full spectrum of macrophage activation. *Nature Reviews Immunology*.

[3] Gaestel M., Kotlyarov A., Kracht M. (2009). Targeting innate immunity protein kinase signalling in inflammation. *Nature Reviews Drug Discovery*.

[4] VanEtten H. D., Mansfield J. W., Bailey J. A., Farmer E. E. (1994). Two classes of plant antibiotics: phytoalexins versus ‘phytoanticipins’. *The Plant Cell*.

[5] Shimizu T., Lin F., Hasegawa M., Okada K., Nojiri H., Yamane H. (2012). Purification and identification of naringenin 7-O-methyltransferase, a key enzyme in biosynthesis of flavonoid phytoalexin sakuranetin in rice. *The Journal of Biological Chemistry*.

[6] Yun J.-M., Im S.-B., Roh M.-K. (2014). Prunus yedoensis bark inhibits lipopolysaccharide-induced inflammatory cytokine synthesis by i*κ*b*α* degradation and MAPK activation in macrophages. *Journal of Medicinal Food*.

[7] Kang H., Kwak T.-K., Kim B.-G., Lee K.-J. (2015). The anti-inflammatory effect of *Prunus yedoensis* bark extract on adipose tissue in diet-induced obese mice. *Evidence-Based Complementary and Alternative Medicine*.

[8] Hernández V., Recio M. C., Máñez S., Giner R. M., Ríos J.-L. (2007). Effects of naturally occurring dihydroflavonols from *Inula viscosa* on inflammation and enzymes involved in the arachidonic acid metabolism. *Life Sciences*.

[9] Toledo A. C., Sakoda C. P. P., Perini A. (2013). Flavonone treatment reverses airway inflammation and remodelling in an asthma murine model. *British Journal of Pharmacology*.

[10] Schroder K., Sweet M. J., Hume D. A. (2006). Signal integration between IFN*γ* and TLR signalling pathways in macrophages. *Immunobiology*.

[11] Abramson S. B., Amin A. R., Clancy R. M., Attur M. (2001). The role of nitric oxide in tissue destruction. *Best Practice and Research: Clinical Rheumatology*.

[12] Hayden M. S., Ghosh S. (2008). Shared principles in NF-*κ*B signaling. *Cell*.

[13] Meraz M. A., White J. M., Sheehan K. C. F. (1996). Targeted disruption of the Stat1 gene in mice reveals unexpected physiologic specificity in the JAK-STAT signaling pathway. *Cell*.

[14] Gao J. J., Filla M. B., Fultz M. J., Vogel S. N., Russell S. W., Murphy W. J. (1998). Autocrine/paracrine IFN-*αβ* mediates the lipopolysaccharide-induced activation of transcription factor stat1*α* in mouse macrophages: pivotal role of stat1*α* in induction of the inducible nitric oxide synthase gene. *Journal of Immunology*.

[15] García-Lafuente A., Guillamón E., Villares A., Rostagno M. A., Martínez J. A. (2009). Flavonoids as anti-inflammatory agents: implications in cancer and cardiovascular disease. *Inflammation Research*.

[16] Pietta P.-G. (2000). Flavonoids as antioxidants. *Journal of Natural Products*.

[17] Bogdan C. (2015). Nitric oxide synthase in innate and adaptive immunity: an update. *Trends in Immunology*.

[18] Kaiko G. E., Horvat J. C., Beagley K. W., Hansbro P. M. (2008). Immunological decision-making: how does the immune system decide to mount a helper T-cell response?. *Immunology*.

[19] Kim S. H., Kim J., Sharma R. P. (2004). Inhibition of p38 and ERK MAP kinases blocks endotoxin-induced nitric oxide production and differentially modulates cytokine expression. *Pharmacological Research*.

[20] Chen C.-C., Tsai P.-C., Wei B.-L., Chiou W.-F. (2008). 8-Prenylkaempferol suppresses inducible nitric oxide synthase expression through interfering with JNK-mediated AP-1 pathway in murine macrophages. *European Journal of Pharmacology*.

[21] Swantek J. L., Cobb M. H., Geppert T. D. (1997). Jun N-terminal kinase/stress-activated protein kinase (JNK/SAPK) is required for lipopolysaccharide stimulation of tumor necrosis factor alpha (TNF-*α*) translation: glucocorticoids inhibit TNF-*α* translation by blocking JNK/SAPK. *Molecular and Cellular Biology*.

[22] Liu G., Xia X.-P., Gong S.-L., Zhao Y. (2006). The macrophage heterogeneity: difference between mouse peritoneal exudate and splenic F4/80^+^ macrophages. *Journal of Cellular Physiology*.

[23] Suttles J., Stout R. D. (2009). Macrophage CD40 signaling: a pivotal regulator of disease protection and pathogenesis. *Seminars in Immunology*.

[24] Qin H., Wilson C. A., Lee S. J., Zhao X., Benveniste E. N. (2005). LPS induces CD40 gene expression through the activation of NF-*κ*B and STAT-1*α* in macrophages and microglia. *Blood*.

[25] Akira S., Takeda K. (2004). Toll-like receptor signalling. *Nature Reviews Immunology*.

[26] Kolb J. P., Casella C. R., Gupta S. S., Chilton P. M., Mitchell T. C. (2014). Type I interferon signaling contributes to the bias that Toll-like receptor 4 exhibits for signaling mediated by the adaptor protein TRIF. *Science Signaling*.

